# Bacillus Calmette-Guérin Vaccine-Induced Lupus Vulgaris in a Child with Bronchial Asthma

**DOI:** 10.5826/dpc.1104a66

**Published:** 2021-10-01

**Authors:** Abdullah Sabra Abualiat, HalaElbedry Edris, Omer Elgaili Yousif Elhag, Ahmed Helmy Nassar, Sayed Agha Shah, Salaheldin Ahmed Alfadni

**Affiliations:** 1 Department of Dermatology and Venereology, Armed Forces Hospitals-Southern Region (AFHSR), Saudi Arabia; 2 Al Neelain University, Faculty of Medicine, Khartoum, Sudan; 3 Department of Dermatology and Venereology, Tanta University, Tanta, Egypt; 4 Department of Pathology, AFHSR and King Khalid University, Saudi Arabia

## Introduction

Lupus vulgaris (LV) is a form of cutaneous tuberculosis (TB) and a rare complication of Bacillus Calmette-Guérin (BCG) vaccination. Although LV is the most common form of cutaneous tuberculosis encountered in adults, it is rare in children. BCG-induced LV is even rarer, with an estimated risk of 5/1,000,000 or 1/100,000 to 175,000 [1]. LV is a paucibacillary form of TB. Therefore, acid-fast bacilli might not be detected in most cases. The diagnosis of cutaneous tuberculosis is based on clinical features, demonstration of acid-fast bacilli on smear, tissue culture, skin biopsy, and in recent years, PCR. However, culture and PCR is often non-conclusive, and diagnoses ought to depend on clinical features, histopathological findings, and the response to treatment [2].

## Case Presentation

**Figure 1:**
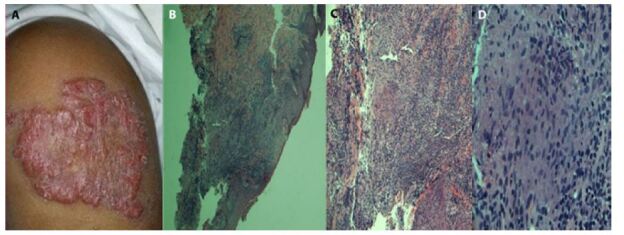
(A) Well-demarcated erythematous scaly heart-shaped plaque in the deltoid area at presentation. (B)Skin punch biopsy shows mild acanthosis and mixed dermal inflammatory cells (magnification ×4). (C) Sections show mixed dermal infiltrate consisting of lymphocytes, epithelioid histiocytes (magnification ×10). (D) Sections show aggregate of epithelioid histiocytes forming granuloma with focal necrosis (magnification ×40).

A 3-year-old boy, an already known case of bronchial asthma, was brought to the dermatology clinic with asymptomatic skin lesions on his left upper arm. The lesion was first observed by themother 2years before presentation, starting as a small lesion, then gradually increasing in size. There were no associated cough, fever, weight loss, or night sweating with a negative family history of a similar condition. The patient received BCG vaccine after birth, then completed the vaccine schedule up to his age. Clinical examination showed a well-demarcated erythematous scaly heart-shaped plaque with a gelatinous consistency on the left deltoid area (site of BCG vaccine) (Figure 1A). The provisional differential diagnosis included cutaneous leishmaniasis, sarcoidosis, and skin tuberculosis.

Histological examination of the punch biopsy revealed the presence of hyperkeratosis, focal parakeratosis, mildly thickened epidermis, and dense lymphohistiocytic infiltrate in the papillary and reticular dermis, accompanied by rare multinucleated giant cells forming tuberculoid granuloma which present interstitially and around vessels with focal necrosis and no LD bodies (Figure 1B–D).

**Figure 2:**
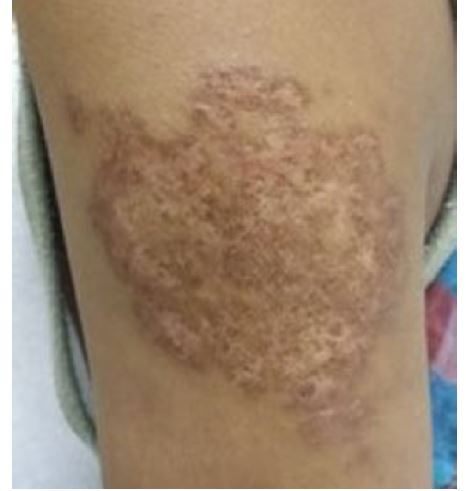
A completely healed lesion with scarring and dyspigmentation at week 8

Apart from high ESR, his lab workup was normal. Screening for an extracutaneous focus of TB was negative. The lesion was treated with an 8-week course of directly observed therapy (DOT) including isoniazid 5mg/kg/week, rifampicin 10 mg/kg/week, pyrazinamide 25 mg/kg/week, and ethambutol 18mg/week, followed by isoniazid and rifampicin for other 16 weeks of therapy. The lesion showed a prompt response to the anti-tuberculous medications and completely healed by the eighth week with moderate residual scarring and dyspigmentation (Figure 2).

## Conclusion

To avoid misdiagnosis, LV should be considered in differential diagnosis, especially in patients with a history of BCG vaccination or any type of tuberculosis with an indicative clinical presentation. A trial of anti-tuberculous drugs could be a diagnostic method when the clinical presentation is highly suspicious where other tests were non-conclusive. In our reported case, LV diagnosis was based on typical clinical and histological findings, together with the dramatic response to anti-tuberculous therapy [2].

Tuberculosis is still an annoying problem in underdeveloped and developing countries due to poor hygiene and low socioeconomic level. Nevertheless, even in developed countries, dermatologists should be aware of the diagnosis of all types of cutaneous tuberculosis, namely post-immunization LV, as it can be easily missed.
